# Recommendations for improved reproducibility of ADC derivation on behalf of the Elekta MRI-linac consortium image analysis working group

**DOI:** 10.1016/j.radonc.2023.109803

**Published:** 2023-07-10

**Authors:** Anne L.H. Bisgaard, Rick Keesman, Astrid L.H.M.W. van Lier, Catherine Coolens, Petra J. van Houdt, Alison Tree, Andreas Wetscherek, Paul B. Romesser, Neelam Tyagi, Monica Lo Russo, Jonas Habrich, Danny Vesprini, Angus Z. Lau, Stella Mook, Peter Chung, Linda G.W. Kerkmeijer, Zeno A.R. Gouw, Ebbe L. Lorenzen, Uulke A. van der Heide, Tine Schytte, Carsten Brink, Faisal Mahmood

**Affiliations:** a Laboratory of Radiation Physics, Department of Oncology, Odense University Hospital, Kløvervænget 19;; b Department of Clinical Research, University of Southern Denmark, J.B. Winsløws Vej 19.3, 5000 Odense Denmark;; c Department of Radiation Oncology, Radboud University Medical Centre, P.O. Box 9101, 6500 HB Nijmegen;; d Department of Radiotherapy, University Medical Centre Utrecht, Heidelberglaan 100, 3584 CX,Utrecht, The Netherlands;; e Department of Medical Physics, Princess Margaret Cancer Centre, University Health Network, 610 University Avenue, M5G 2M9 Toronto, ON, Canada;; f Department of Radiation Oncology, the Netherlands Cancer Institute, Postbus 90203, 1006 BE Amsterdam, The Netherlands;; g Department of Urology, The Institute of Cancer Research and The Royal Marsden NHS Foundation Trust, Downs Road, Sutton, Surrey, SM2 5PT London;; h Joint Department of Physics, The Institute of Cancer Research and The Royal Marsden NHS Foundation Trust, SM2 5NG London, UK;; i Department of Radiation Oncology, Memorial Sloan Kettering Cancer Center, 1275 York Avenue, Box 22, NY 10065;; j Department of Medical Physics, Memorial Sloan Kettering Cancer Center, 545 E. 73rd street, NY 10021, New York, USA;; k Department of Radiation Oncology, University Hospital and Medical Faculty, Eberhard Karls University;; l Section for Biomedical Physics, Department of Radiation Oncology, University of Tübingen, Hoppe-Seyler-Str. 3, 72076 Tübingen, Germany;; m Department of Radiation Oncology, Sunnybrook Odette Cancer Centre;; n Physical Sciences Platform, Sunnybrook Research Institute. Department of Medical Biophysics, University of Toronto, 2075 Bayview Avenue, M4N 3M5 Toronto;; o Radiation Medicine Program, Princess Margaret Cancer Centre, University Health Network. Department of Radiation Oncology, University of Toronto, 610 University Avenue, M5G 2M9 Toronto, ON, Canada;; p Department of Oncology, Odense University Hospital, Kløvervænget 19, 5000 Odense, Denmark

**Keywords:** Apparent diffusion coefficient, MRI-Linac, Adaptive radiotheray, Diffusion-weighted magnetic resonance imaging, ADC reproducibility, MRI biomarkers

## Abstract

**Background and purpose:**

The apparent diffusion coefficient (ADC), a potential imaging biomarker for radiotherapy response, needs to be reproducible before translation into clinical use. The aim of this study was to evaluate the multi-centre delineation- and calculation-related ADC variation and give recommendations to minimize it.

**Materials and methods:**

Nine centres received identical diffusion-weighted and anatomical magnetic resonance images of different cancerous tumours (adrenal gland, pelvic oligo metastasis, pancreas, and prostate). All centres delineated the gross tumour volume (GTV), clinical target volume (CTV), and viable tumour volume (VTV), and calculated ADCs using both their local calculation methods and each of the following calculation conditions: b-values 0–500 vs. 150–500 s/mm^2^, region-of-interest (ROI)-based vs. voxel-based calculation, and mean vs. median. ADC variation was assessed using the mean coefficient of variation across delineations (CV_D_) and calculation methods (CV_C_). Absolute ADC differences between calculation conditions were evaluated using Friedman’s test. Recommendations for ADC calculation were formulated based on observations and discussions within the Elekta MRI-linac consortium image analysis working group.

**Results:**

The median (range) CV_D_ and CV_C_ were 0.06 (0.02–0.32) and 0.17 (0.08–0.26), respectively. The ADC estimates differed 18% between b-value sets and 4% between ROI/voxel-based calculation (p-valu es < 0.01). No significant difference was observed between mean and median (p = 0.64). Aligning calculation conditions between centres reduced CV_C_ to 0.04 (0.01–0.16). CV_D_ was comparable between ROI types.

**Conclusion:**

Overall, calculation methods had a larger impact on ADC reproducibility compared to delineation. Based on the results, significant sources of variation were identified, which should be considered when initiating new studies, in particular multi-centre investigations.

Quantitative imaging biomarkers (QIBs), derived from in-vivo imaging, are useful in oncology, as they non-invasively provide quantitative information on tissue characteristics [1–3]. Development of QIBs has the potential to improve precision and reduce subjectivity of image analysis, and hereby enable a more robust association between image-derived parameters and biological and clinical parameters [4,5]. QIBs may provide spatially and temporally resolved information linked to tumour biology, which in radiotherapy may be used for improved target delineation, dose-painting and prediction and monitoring of response. Hence, QIBs may improve personalization of the treatment [6].

The advanced magnetic resonance imaging (MRI) technique, diffusion-weighted MRI (DWI), is a potential QIB for the above-mentioned radiotherapy purposes [6–9]. In standard DWI, strong magnetic gradients are applied to sensitize the MRI signal to the random motion of water molecules within the scanned object. The amount of diffusion weighting is defined by the b-value, and if at least two appropriately selected b-values are acquired, the quantitative parameter, the apparent diffusion coefficient (ADC), can be derived. ADC correlates with tissue cellularity, and have been shown to identify *radio-resistant* regions [10,11]. DWI and derived ADC maps are used in the clinic to guide target delineation for some tumours, and may be a future tool for dose painting [12,13]. Further, baseline ADC and ADC changes during treatment have shown potential to predict response, although lack of consistency is preventing translation to the clinic [8,14–17]. Specifically, varying acquisition protocols and analysis methods reduce ADC reproducibility, potentially hindering validation of ADC as a QIB. To overcome this problem, a standardization of measurements is needed, and large multi-centre validation trials are warranted [2,18].

Hybrid MRI linear accelerators (MRI-linac) allows daily measurement of ADC, with no or limited prolongation of the radiotherapy fractions [19,20]. As such, an MRI-linac provides an ideal platform for clinical validation of potential QIBs such as ADC. Accuracy of ADC on MRI-linac has been demonstrated using phantoms, and feasibility has been demonstrated in patients [18,21,22]. Furthermore, recommendations for MRI protocols to acquire DWI on an Elekta MRI-linac have been published [23]. The current study focused on the analysis of the acquired DWI scans to obtain an ADC value.

Different approaches to DWI analysis may introduce a variation across centres/studies. Within the Elekta MRI-linac consortium image analysis working group [24], two expected sources of variation were identified: The delineation of a region of interest (ROI), and the calculation method. Delineation uncertainty is a well-known source of uncertainty in radiotherapy and propagates as ADC variation as well [25,26]. The impact of calculation methods on ADC reproducibility has been investigated to a lesser extent [27]. The current study investigated the impact of variations in both delineations and calculation methods on the ADC reproducibility utilizing the same data, which enabled assessment of their relative contributions. The aim was to give vendor-neutral recommendations to improve ADC reproducibility, based on an evaluation of the observed ADC variation between MRI-linac centres and discussions within the working group.

## Methods

### Study design

**Fig. 1.**
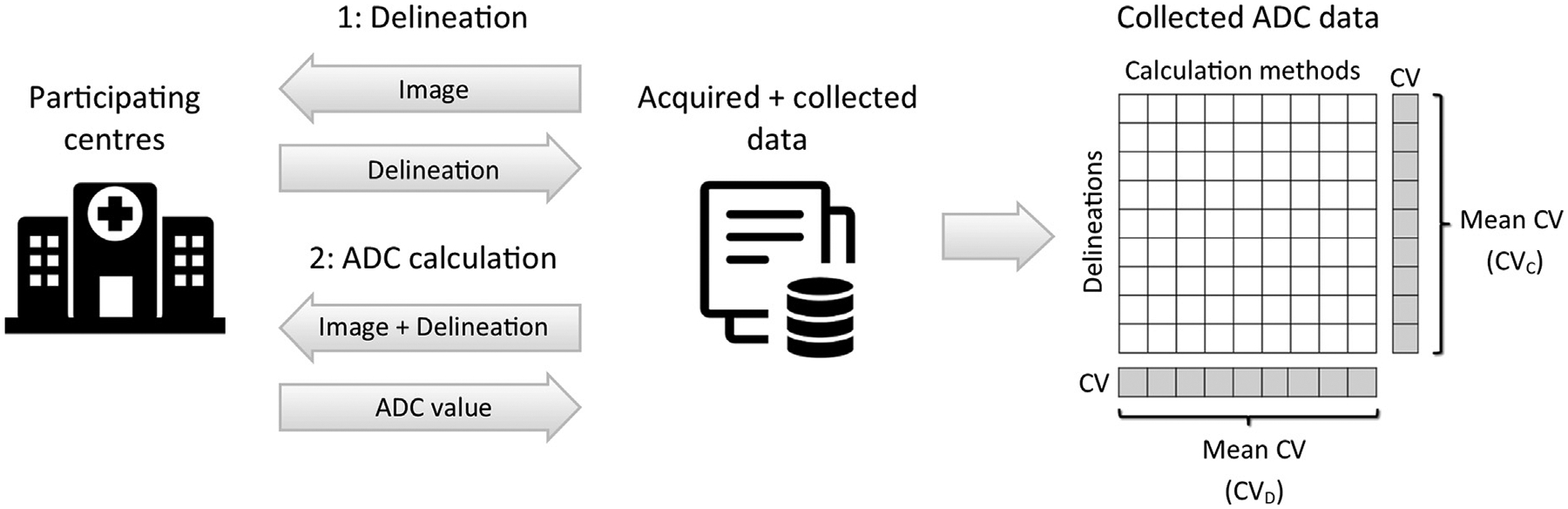
Study design. Each of the nine participating centres performed delineation and ADC calculation. The collected ADC values were organized in a table as illustrated to the right, where rows and columns represent the delineations and calculation methods from the nine centres. Tables were made for each combination of cancer diagnosis and delineation types (GTV, CTV, VTV). The ADC variation across delineations and calculation methods were assessed using the mean coefficient of variation (CV), as indicated on the table.

Nine MRI-linac centres participated in the study using anonymized patient MRI data from four different clinical cases, acquired at one of the participating centres. At each centre, two steps were performed (Fig. 1). In step 1, an oncologist performed delineations. In step 2, each centre calculated ADC for delineations made at all centres using their local calculation method. This resulted in a 9×9 table of ADC values for each clinical case and delineation type.

### Clinical cases

The study included four patients with different cancerous tumours.

1. Adrenal gland (76 year old male with oligo progression after systemic treatment for non-small cell lung cancer)
2. Pancreas (68 year old male with recurrent pancreas cancer, consolidative radiotherapy after systemic treatment)
3. Oligo metastasis in the pelvis (54 year old woman with recurrent ovarian cancer, consolidative radiotherapy after systemic treatment)
4. Prostate and adjacent seminal vesicles (74 years old man with low volume metastatic prostate cancer)

All patients received treatment on the same 1.5 T MRI-linac (Unity by Elekta, Stockholm, Sweden) at one of the participating centres. The patients were included in the MOMENTUM study (clinicaltrials.gov NCT04075305) [28]. Informed consent was obtained from all patients, and DICOM-data was anonymized and stored adhering to ethics standards.

### MRI data

MRI data were acquired at fraction one, prior to beam delivery and included T2-weighted images (T2W) and DWI with the b-values 30, 80, 150, 300 and 500 s/mm^2^ (adrenal gland and pancreas), and 0, 30, 80, 150 and 500 s/mm^2^ (oligo metastasis and prostate) adhering to the normal MRI-linac workflow [29]. Sequence details are listed in Table S1 in supplementary materials. DWI were acquired twice in succession while the patient remained in position, to obtain test–retest data for repeatability estimation.

### Delineation

T2W images and DWI images with b-values 150 and 500 s/mm^2^ were available for delineation. Provided with brief clinical case descriptions, the oncologists delineated the gross tumour volume (GTV), clinical target volume (CTV) (prostate only) and the viable tumour volume (VTV) (except for prostate) in a mutually blinded manner using the ProKnow platform (Version 1.32.0, Elekta Solutions AB, Stockholm, Sweden). The VTV was defined as the GTV excluding cystic and necrotic parts. A description of the technical data preparation is given in supplementary materials.

### ADC calculation

Each centre provided a brief description of their local calculation method, including software implementation, choice of b-values, and whether a ROI- or voxel-based calculation was used. The ROI-based method refers to ADC calculation using the mean or median ROI signals of DWIs, whereas the voxel-based method refers to calculating ADC within each voxel, after which the mean or median value is calculated within the ROI. If a centre’s standard approach was to use the scanner software for ADC calculation, that centre was provided with ADC maps calculated with the scanner software using all b-values, the lowest and the highest value, and b ≥ 150 s/mm^2^, respectively. They were asked to choose the set best representing their normal choice.

Each centre provided ADC values for both their own and other centre’s delineations. The calculation was based on 1: the centre’s own calculation method, and 2: all combinations of the following calculation conditions: all b-values vs. b ≥ 150 s/mm^2^, ROI-based vs. voxel-based and mean vs. median (referred to as pre-specified calculation conditions).

### Data analysis and statistics

Delineations were compared pairwise to calculate the Dice similarity coefficient (Dice) and mean surface distance (MSD). ADC variation across delineations and calculation methods was assessed using the mean coefficient of variation (CV), calculated in the following way (Cf. Fig. 1): The CV describing variation across calculation methods was calculated for each of the nine delineations, and the average of these nine values was used as a measure of variation across calculation methods (CV_C_). Likewise, the CV describing variation across delineations was calculated for each of the nine calculation methods, and the average was used as a measure of variation across delineations (CV_D_).

Retest ADC values were calculated using rigid contour propagation of GTVs between test- and retest-scans. Median ADC values within the GTVs were extracted from ADC maps calculated with the scanner software using b ≥ 150 s/mm^2^. The within-subject coefficient of variation (wCV) was calculated as a measure of test–retest ADC variation (ADC repeatability), as recommended by the Quantitative Imaging Biomarkers Alliance (QIBA) [30].

The ADC difference between the sets of b-values, ROI/voxel-based analysis and mean/median values, respectively, were evaluated using Friedman tests with a 5 % significance level and with Bonferroni correction for multiple testing. Only GTVs were used for this purpose.

## Results

A total of 69 out of 72 expected delineated volumes (9 centres × 8 volumes) were available for the analysis. Within these volumes, a total of 4483 ADC values were obtained out of 5589 (69 delineation × 9 centres × 9 combinations of calculation conditions). The reasons for the reduced number were the following: One centre omitted calculation within two prostate volumes and two centres omitted the ROI-based calculations due to technical difficulties or limitations of their local software. One centre omitted ADC calculation using the pre-specified calculation conditions due to limited time and resources. One centre used software that reported only one decimal, which in some cases led to CV’s of zero. CV’s of zero were excluded before calculating the mean CV.

**Fig. 2.**
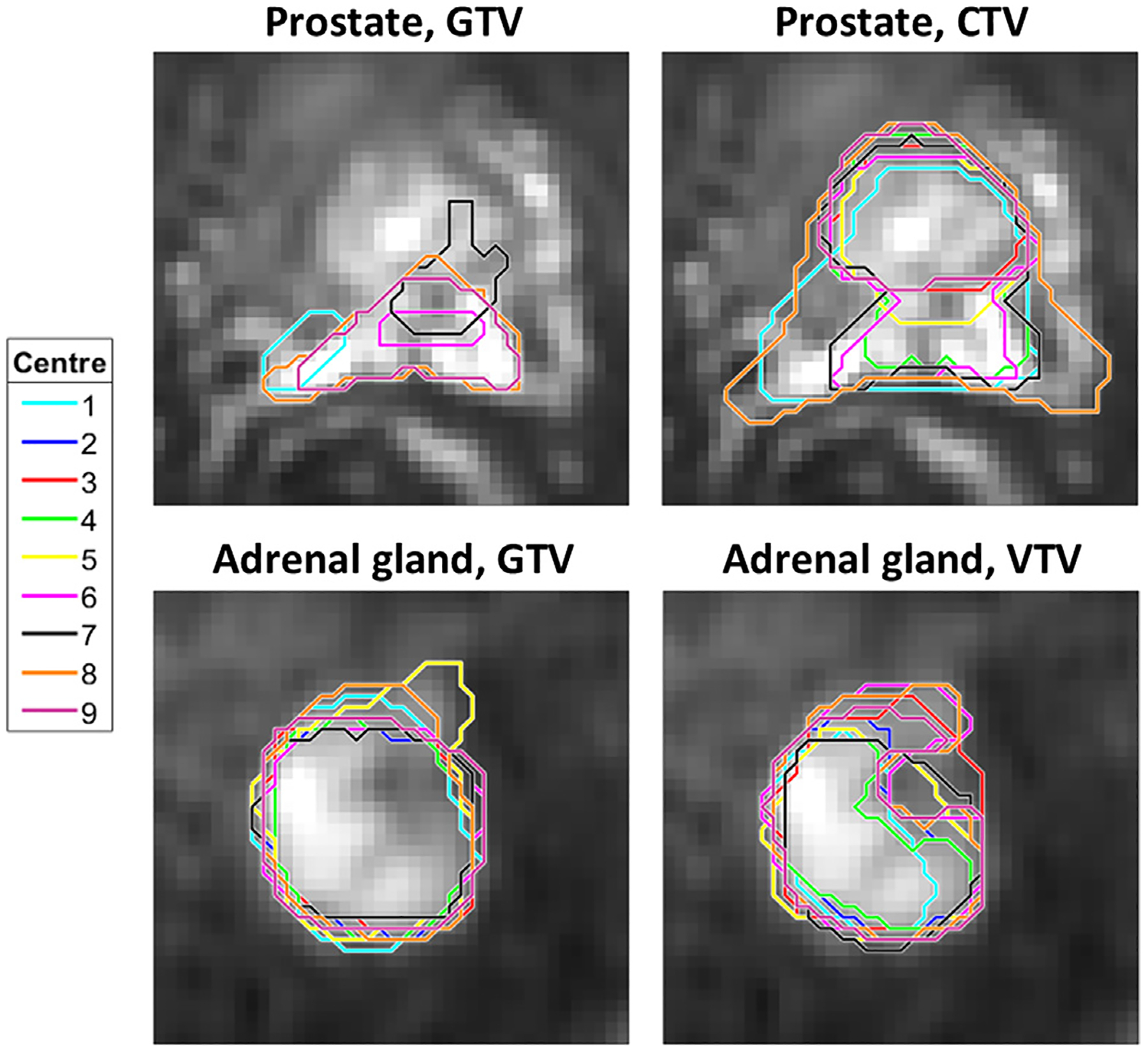
Examples of delineations. Delineations made by the nine participating centres for prostate and adrenal gland, shown on b = 500 /mm^2^ DWI images, cropped to an area of 7.7 × 7.7 cm^2^ (prostate) and 4.9 × 4.9 cm^2^ (adrenal gland) around the tumour. For the prostate, not all delineated contours included the shown slice, thus, only five contours are visible.

**Fig. 3.**
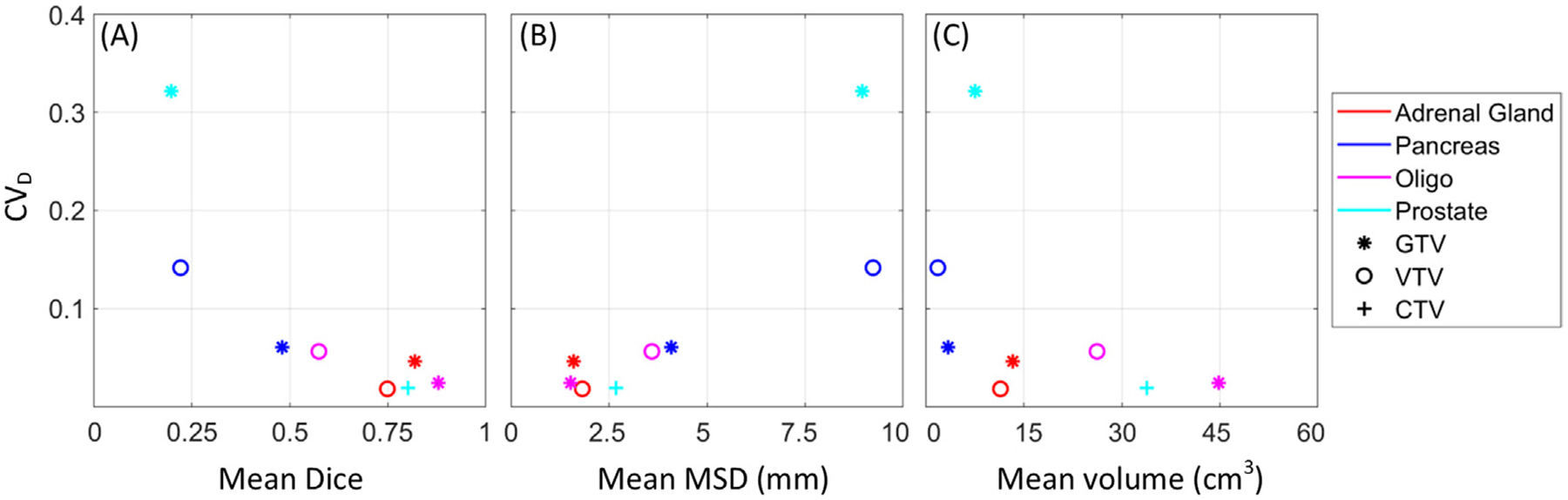
Delineation-related ADC variation. Delineation-related ADC variation (mean coefficient of variation, CV_D_) as a function of mean Dice Similarity Coefficient (A), Mean Surface Distance, MSD (B), and volume (C), for the different clinical cases (marker colors) and types of ROIs (marker types).

Representative delineations are presented in Fig. 2. The delineation variation was large for pancreas VTV and prostate GTV (Dice: 0.20–0.22 and MSD: 9.09–9.23 mm) compared to the remaining cases (Dice: 0.48–0.88 and MSD: 1.52–4.09 mm) (Fig. 3.A–B). A closer inspection of the prostate delineations revealed that some GTV delineations did not overlap (Fig. 2). The prostate CTV delineation variation was smaller (Dice: 0.80, MSD: 2.68 mm), despite not all centres included the seminal vesicles in the delineation. The CV_D_ was comparable between GTV and VTV, although the delineation variation was slightly smaller for GTV compared to VTV (Fig. 3.A–B). There was a clear correlation between delineation variation and ADC variation (Fig. 3.A–B).

All centres used a voxel-based approach as their local calculation method. One centre used ADC maps generated by the scanner software, while remaining centres used in-house software for ADC calculation with a mono-exponential Stejskal-Tanner model [31]. The main differences between the local calculation methods were the choice of b-values, fitting method, and applied filtering. A full comparison of the centres’ local calculation methods is presented in Table S2 in supplementary materials.

**Fig. 4.**
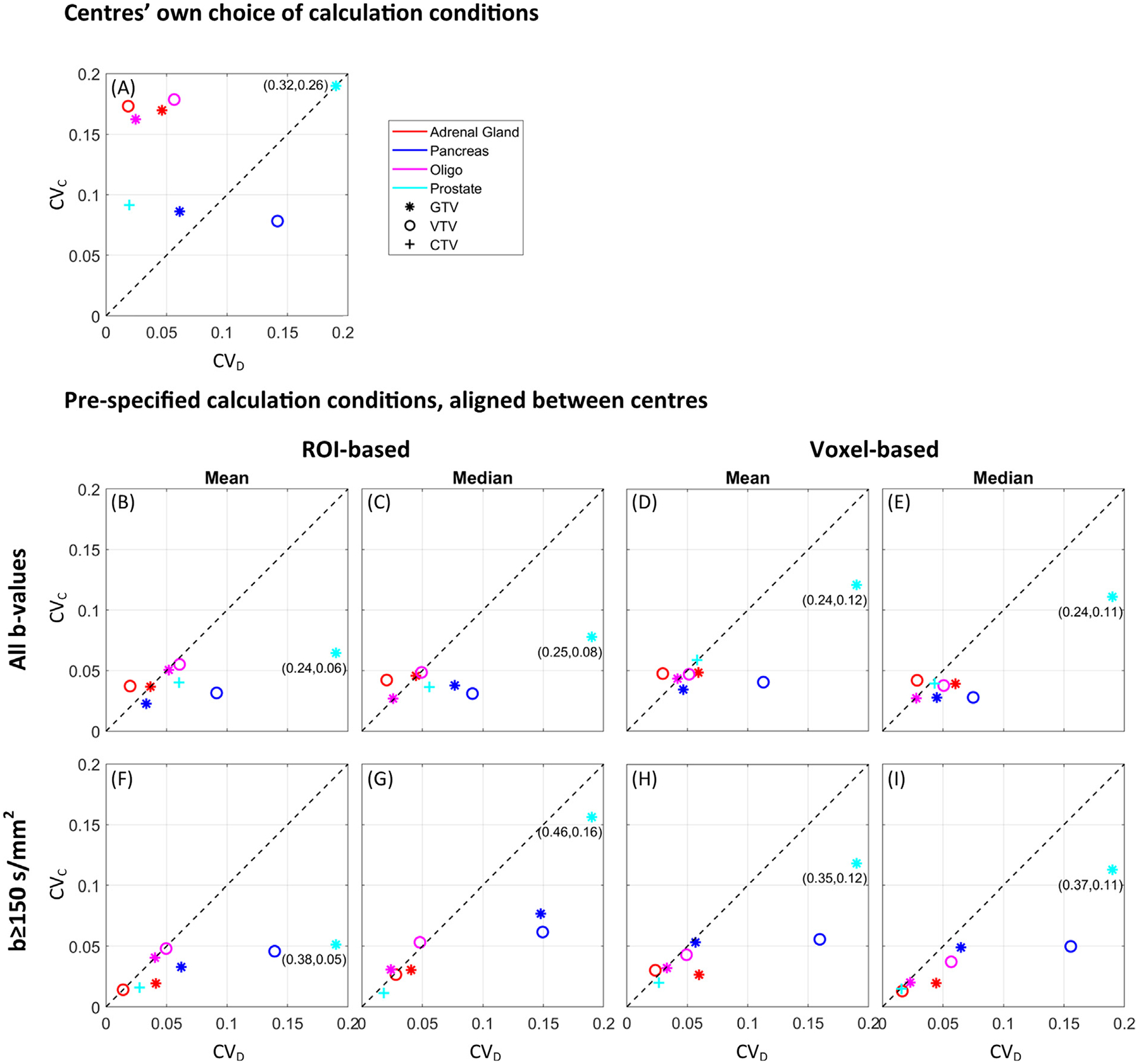
ADC variation. ADC mean coefficient of variation across delineations (CV_D_) and calculation methods (CV_C_) from the nine MRI-linac centres, with the centres’ own choice of calculation conditions (A), and with pre-specified calculation conditions (B-I). Median ADC values were used in (A). The marker colours and types represent the different clinical cases and types of ROIs. The dotted line at x = y represents the points where delineation- and calculation-related ADC variation are the same. For the prostate GTV, CV_D_ is outside the axis range, and therefore, the true coordinates are indicated next to the marker.

With the centres’ own calculation methods, the median (range) CV_D_ and CV_C_ were 0.06 (0.02 – 0.32) and 0.17 (0.08 – 0.26), respectively (Fig. 4.A). The delineation-related variation was larger for pancreas VTV and prostate GTV (CV_D_: 0.15–0.32) compared to the remaining cases (CV_D_: 0.02–0.06). In comparison, the ADC repeatability (wCV) based on test–retest scans was estimated to 4.0% (adrenal gland), 6.6% (pancreas), 1.3% (oligo metastasis), and 15.2% (prostate). A detailed overview of the ADC variation for each delineation and calculation method is shown in Fig. S1–3 in supplementary materials.

When centers aligned their calculation methods according to any of the pre-specified calculation conditions, the calculation-related ADC variation was clearly smaller than when centres used their own choice of calculation conditions (Fig. 4.B–I compared to Fig. 4.A), with a reduction of median (range) CV_C_ to 0.04 (0.01–0.16) (or 0.04 (0.01–0.08) with the low-agreement prostate GTV excluded).

In terms of absolute ADC, there was a trend towards larger values for calculation methods that included b-values below 150 s/mm^2^, (calculation methods no. 1, 4 and 9 in Figure S1 and S3 and Table S2 in supplementary materials). Averaged across all combinations of the pre-specified calculation conditions, ADC estimates were 18% larger for the full b-set compared to b ≥ 150 s/mm^2^ (p < 0.01) and 4% larger for ROI-based analysis compared to voxel-based (p < 0.01) (Table 1). There was no significant difference between mean and median values (p = 0.64).

## Discussion

This study evaluated the ADC variation related to differences in delineation and calculation methods between centres. The calculation-related variation was generally larger than delineation-related variation (Fig. 4.A), and was primarily driven by different choices of b-values. When calculation conditions (all b-values vs. b ≥ 150 s/mm^2^, ROI-based vs. voxel-based, and mean vs. median) were aligned between centres, the calculation-related variation was reduced to about the same level as the delineation-related variation. Furthermore, the delineation- and calculation-related ADC variation was comparable to the ADC repeatability, indicating that acquisition and post-processing of the images contribute equally to the ADC variation. The GTV and VTV performed comparably with respect to ADC reproducibility.

Overall, the observed delineation-related ADC variation largely agreed with other studies, showing CV of 0.1 and inter-observer coefficient of repeatability of 1.9–14% in pancreas [32,33], and 9.5–13.7% in prostate [34], although not directly comparable due to differences in methods. The large delineation variation of the pancreas VTV was likely due to the higher sensitivity to delineation of small volumes (Fig. 3.C). For the prostate GTV, the large delineation variation could arise from the GTV not being a standard delineation type. In fact, large variation in definitions of intraprostatic lesions has been reported in earlier studies [35,36]. Potentially, the use of a higher b-value would have improved the conspicuity of the intra-prostatic lesions. To comply with the MRI-linac recommendations, a maximum b-value of 500 s/mm^2^ was used [18,23]. The delineation variation in prostate may also have been overestimated as not all centres included the vesicles in the CTV (as case descriptions indicated).

Other studies have shown that the type of ROI influences both absolute ADC values, relative ADC changes during treatment, and the reproducibility of delineations [18,25,26,37]. Therefore, this study included two types of ROIs. The GTV, because it has the advantage of being available before the start of treatment in both the standard and MRI-linac radiotherapy workflow. The so-called VTV was included because it excludes non-viable parts of the tumour and may be relevant for probing the cellular response directly and assessing treatment response, as suggested by Padhani et al. [18]. Further, one study showed that ADC based on VTV was superior to GTV in stratifying between responding and non-responding patients [38]. An advantage of the VTV is that, by definition, it contains only high signal-to-noise-ratio (SNR) voxels. For tumours with no significant necrotic/cystic components, e.g. prostate, the VTV corresponds to the GTV.

Since the choice of ROI type did not influence the ADC reproducibility in the current study, selection of ROI type depends on its application in radiotherapy. While the VTV may define radio-resistant regions and be relevant for dose painting, it is not obvious which ROI is best suited for response prediction. The literature investigating the potential benefits of using GTV vs. VTV is limited [16,38]. In general, the results of the current study advocate improving delineation consistency (Fig. 3.A–B), which underlines the importance of having as precise consensus guidelines as possible. In the future, delineation variation may be reduced by automatic delineation tools including AI models, as indicated in several studies [17,39–41].

The DWI-signal is sensitive to perfusion at low b-values (below 100 s/mm^2^), and therefore, including low b-values in the analysis is expected to overestimate ADCs [18,42] as observed in this study also (all b-values compared to b ≥ 150 s/mm^2^) (Table 1). Therefore, a previous publication by the Elekta MRI-linac working group, recommended that the lowest b-value should be 100–150 s/mm^2^ [23]. A maximum b-value of 500 s/mm^2^ was also recommended to ensure sufficient SNR and a diffusion time comparable to that of a diagnostic scanner. Moreover, if notably higher b-values are included in the calculation (b > 1000 s/mm^2^), non-Gaussian diffusion effects may result in an underestimation of ADC, as the mono-exponential model assumes a Gaussian diffusion behaviour [43].

The ROI- and voxel-based approach have been used in previous studies and are therefore relevant from a reproducibility point-of-view [25,33,37,44,45]. It should be noted that strictly speaking, the average ADC across voxels within a ROI cannot be derived using the ROI-based approach, which is based on the mean DWI signal within the ROI. I.e. the ROI-based method is mathematically inconsistent with the exponential model of ADC calculation (when more than one voxel is present within a ROI). However, using the ROI-based method may lead to better estimates of ADC as it is expected to be more robust to motion induced misalignment of individual DWI acquired at different b-values, which if not properly corrected can lead to invalid ADC values. Further, it may improve SNR which may give a better goodness of fit of data, as was confirmed using the current data (not shown) [6]. In the current study, the ROI-based approach led to larger ADC values compared to the voxel-based approach (Table 1), while the two approaches performed comparable with respect to ADC reproducibility (Fig. 4.B–I).

The residual calculation-related ADC variation present after aligning the pre-specified calculation conditions between centres (Fig. 4.B–I) may be accounted for as use of different software implementations, including different fitting and filtering methods (Table S2 in supplementary materials). Specifically, five centres used linear least squares fitting of ln(S) as a function of b-values to estimate the ADC (Table S2 in supplementary materials). Since the SNR decreases with increasing b-value, the uncertainty of ln (S) also increases with b-values, if not accounted for by averaging signals from multiple excitations. Thus, a better approach will be to use weighted linear least squares fitting (see supplementary materials) [46]. For the voxel-based approach, five centres used filtering by excluding voxels containing non-physical values, i.e. values outside a certain range (Table S2 in supplementary materials). Alternative to this, voxels may be removed based on low SNR or poor quality of the fit, which is a more objective criterion. Contributions from fitting and filtering were not determined individually, nevertheless, in combination, they resulted in calculation-related ADC variations comparable to the delineation-related variations (points close to the dotted line in Fig. 4.B–I). This stresses the importance of excluding sources of variation whenever possible, especially if the aim is to establish common ADC cut-off values, e.g. for response prediction. Making a platform-independent software available for public download might be a way to proceed such that in-house developed software can be validated against a common software.

The SNR has also been shown to play a role in estimation of the ADC [23,47]. Although not specifically investigated in this study, it is worth mentioning a few implications. Low SNR levels lead to an underestimation of the ADC, due to the so-called noise floor present in magnitude reconstructed DWI-images [6,48]. Therefore, to allow a comparison between studies, the SNR should always be reported based on defined standards, e.g. published by the National Electrical Manufacturers Association (NEMA) [49] or QIBA [50]. For practicality, it may be sufficient to measure SNR once, if patient and coil positioning is consistent between scans [6]. Applying noise correction has been shown to reduce the ADC bias [47].

Other specific points of attention when calculating ADC include pre-processing of the image data. For example, to minimize the effect of motion, registration between b-values is recommended [51], and is available on most MRI scanner software, including the Unity MRI-linac. As a minimum, b-value images should visually be inspected for motion and artefacts. Further, as the intensity-histogram of DWI images may be “stretched” to fully utilize the storage bit depth, the stored pixel values should as a general rule be “unscaled” prior to quantitative analysis as described by Chenevert et al. [52].

A main limitation of this study is that only one patient was included per tumour type. This was deemed a necessary compromise to increase the realizability of the investigation. However, by including four tumour types instead of e.g. four tumours of the same type, we were able detect differences in the analysis-related ADC variation between tumour types. Minor limitations include that no re-positioning of the patient was performed between the test and retest scans, which may underestimate the true repeatability. ADC reproducibility may also be affected by the sequence used to acquire the images (turbo-spin-echo (TSE) vs. echo planar imaging (EPI) [53]) and the diffusion time [54], but investigations of this was outside the scope of the current study where EPI based readout was used. Moreover, as EPI is notorious for low geometric accuracy [55], a high ADC reproducibility can still lead to a misinterpretation of the extent of the GTV. The effect of geometric distortions on ADC reproducibility and GTV misalignment should be investigated in a future study.

## Conclusion

This investigation provides recommendations for improving reproducibility of ADC calculations, based on observations and discussions within the Elekta MRI-linac consortium image analysis working group. These recommendations are focused towards future investigations of ADC as a potential imaging biomarker in radiotherapy. Investigations of other potential quantitative imaging biomarkers using a similar setup, and the geometric accuracy of these, are warranted.

In summary, the calculation-related ADC variation was larger than the delineation-related ADC variation. Specifically, the calculation-related ADC variation can be attributed to the choice of b-values, ROI-based/voxel-based calculation, and software implementation including fitting and filtering method. Therefore, it is recommended to align these factors in multi-centre studies, and to report details of the ADC calculation method within a study to allow comparison between studies. In general, delineation variation correlates with ADC variation, and should therefore be reduced as much as possible. Selection of GTV vs. a dedicated volume for ADC derivation seems less critical for ADC reproducibility, and should depend primarily on feasibility and the radiotherapy purpose.

## Supplementary Material

Supplementary

## Figures and Tables

**Table 1:** Mean ADC values (x 10^−3^ mm^2^/s) across nine centres for different combinations of calculation conditions. The mean %-wise ADC differences between b-sets (all-b-values minus b≥150 s/mm^2^), ROI/voxel-based analysis (ROI-based minus voxel-based) and mean/median values (mean minus median) are shown.

	All b-values				b ≥ 150 s/mm^2^				Mean difference (%)		
	ROI-based		Voxel-based		ROI-based		Voxel-based				
	Mean	Median	Mean	Median	Mean	Median	Mean	Median	b-sets	ROI/vox	Mean/median
Adrenal gland											
GTV	1.27	1.37	1.21	1.24	0.90	0.97	0.88	0.90	32.86	5.84	−4.91
VTV	1.26	1.34	1.23	1.24	0.88	0.96	0.87	0.90	33.69	4.40	−4.47
Pancreas											
GTV	0.95	0.95	0.88	0.96	0.88	0.85	0.81	0.82	11.05	4.30	−1.59
VTV	1.08	1.08	1.03	1.07	1.03	1.06	0.98	1.00	4.59	3.90	−2.18
Oligo metastasis											
GTV	1.51	1.41	1.44	1.34	1.33	1.33	1.29	1.19	10.35	5.99	5.48
VTV	1.49	1.39	1.43	1.32	1.33	1.31	1.29	1.18	9.58	5.45	6.33
Prostate											
GTV	1.41	1.37	1.35	1.34	1.07	0.99	1.03	1.02	28.41	1.85	3.09
CTV	1.56	1.52	1.49	1.46	1.31	1.29	1.27	1.29	15.62	3.00	1.13
Mean value	1.32	1.30	1.26	1.25	1.09	1.09	1.05	1.04	18.27	4.34	0.36
