# Supplementary material for: Recommendations for improved reproducibility of ADC derivation on behalf of the Elekta MRI-linac consortium image analysis working group
Source: Radiother Oncol. Author manuscript; Available in PMC 2024 Jun 25. (PMC11197850; doi:10.1016/j.radonc.2023.109803)
Supplement: Supplementary [file NIHMS1996513-supplement-Supplementary.docx]

Supplementary materials

# Table S1: MRI sequence details

Table S1: Details on DWI and T2W sequences. Abbreviations: Spin echo (SE), echo planar imaging (EPI), spectral presaturation with inversion recovery (SPIR), spectral attenuated inversion recovery (SPAIR)

|  | **Adrenal gland** | **Pancreas** | **Oligo metastasis** | **Prostate** |
| --- | --- | --- | --- | --- |
| **T2W:** | | | | |
| Sequence | 3D SE | 3D SE | 3D SE | 3D SE |
| Fat saturation | No | No | No | No |
| TE/TR (ms) | 137/1400 | 137/1400 | 151/1400 | 151/1400 |
| Parallel imaging (SENSE factor) | 3.7 | 3.7 | 3.7 | 3.7 |
| No. of excitations (NEX) | 3 | 3 | 2 | 2 |
| In-plane resolution (mm) | 1x1 | 1x1 | 0.81x0.81 | 1x1 |
| Field of view (mm) | 448x448 | 448x448 | 544x544 | 448x448 |
| Slice thickness (mm) | 2 | 2 | 2 | 2 |
| Slice gap (mm) | 0 | 0 | 0 | 0 |
| Scan duration | 6 min and 4 s | 6 min and 4 s | 5 min and 32 s | 3 min and 51 s |
| **DWI:** | | | | |
| Sequence | Monopolar diffusion encoding, 2D SE with single shot EPI readout | Monopolar diffusion encoding, 2D SE with single shot EPI readout | Monopolar diffusion encoding, 2D SE with single shot EPI readout | Monopolar diffusion encoding, 2D SE with single shot EPI readout |
| Diffusion gradient encoding | Three orthogonal directions along the imaging plane axes | Three orthogonal directions along the imaging plane axes | Three orthogonal directions along the imaging plane axes | Three orthogonal directions along the imaging plane axes |
| Fat saturation | SPIR | SPIR | SPAIR | SPAIR |
| b-values (No. of excitations (NEX)) | 30 (2)  80 (2)  150 (4)  300 (4)  500 (16) | 30 (2)  80 (2)  150 (4)  300 (4)  500 (16) | 0 (2)  30 (2)  80 (2)  150 (4)  500 (16) | 0 (2)  30 (2)  80 (2)  150 (4)  500 (16) |
| Gradient duration (ms) ($\delta$) | 22.47 | 22.47 | 20.22 | 20.22 |
| Effective diffusion time (ms) $\left( \Delta-\frac{\delta}{3} \right)$ | 27.95 | 27.95 | 34.40 | 34.40 |
| TE/TR (ms) | 70.90/559.43 | 70.90/559.43 | 82.30/4538.00 | 82.30/3354.17 |
| Parallel imaging (SENSE factor) | 2 | 2 | 2.3 | 2.3 |
| In-plane resolution (mm) | 1.22x1.22 | 1.22x1.22 | 1.92x1.92 | 1.92x1.92 |
| Field of view (mm) | 351x351 | 351x351 | 430x430 | 430x430 |
| Slice thickness (mm) | 6 | 6 | 4 | 4 |
| Slice gap (mm) | 0.6 | 0.6 | 0 | 0 |
| Scan duration | 2 min and 23 s | 2 min and 23 s | 5 min and 40 s | 4 min and 12 s |

# Technical preparation of data

DWI images were split into individual b-value-images with unique series UID’s using in-house software (Matlab R2020b, Mathworks ab, Sweden) to accommodate delineation in ProKnow. For each b-value, a rigid registration between the DWI and T2W image was performed using MIM (MIM Software Inc., Cleveland, Ohio).

The original, non-split DWI images were used for ADC calculation. A registration between b-values was performed using the built-in function of the scanner software (R5.7.1, Philips Ingenia, Philips Healthcare, Best, The Netherlands). All delineations were transferred to the original DWI images using the transfer matrix from the rigid registration between b=500 s/mm^2^ DWI and T2W images in MIM.

# Table S2: Calculation method details

Table S2: Details on calculation methods used by the nine participating centres.

| Calculation method No. | b-values (s/mm^2^) | Fitting method | Filtering | Normally reported ADC metrics |
| --- | --- | --- | --- | --- |
| 1 | All | Linear least squares | Values below 10^-6^ mm^2^/s were excluded | Histogram distribution. Median (range). Mean |
| 2 | $\geq$150 | Linear least squares | No | Median + 25th and 75th percentiles |
| 3 | $\geq$150 | Linear least squares | Values below 0 mm^2^/s were excluded | Median |
| 4 | Adrenal gland + pancreas: 30, 80, 150, 300, 500  Oligo metastasis + prostate: 0, 80, 150, 500 | Linear least squares | Values below 0 mm^2^/s were excluded | Mean |
| 5 | 150 and 500 | Direct solution (no fit):  ADC[mm^2^/s]= -ln(S(500)/S(150))/ (500-150) | Limiting ADC values to range  (0-4) × 10^-3^ mm^2^/s. Values were not excluded. | Mean + SD |
| 6 | $\geq$150 | Scanner software | No | Mean+SD (or Median + 5^th^/95^th^ percentile if clinical software provided those) |
| 7 | $\geq$150 | Linear least square | Values below 0 mm^2^/s were excluded | Median ADC + 25th and 75th percentiles |
| 8 | $\geq$150 | Non-linear least squares | Lower bound of 0 and upper bound of 3.1×10^-3^ mm^2^/s | Mean ADC for GTV. |
| 9 | All | Weighted linear regression on function  Weighting function: 1/S(b) | No | Change in median ADC + 75th percentile |

# Figure S1-3: ADC variation boxplots


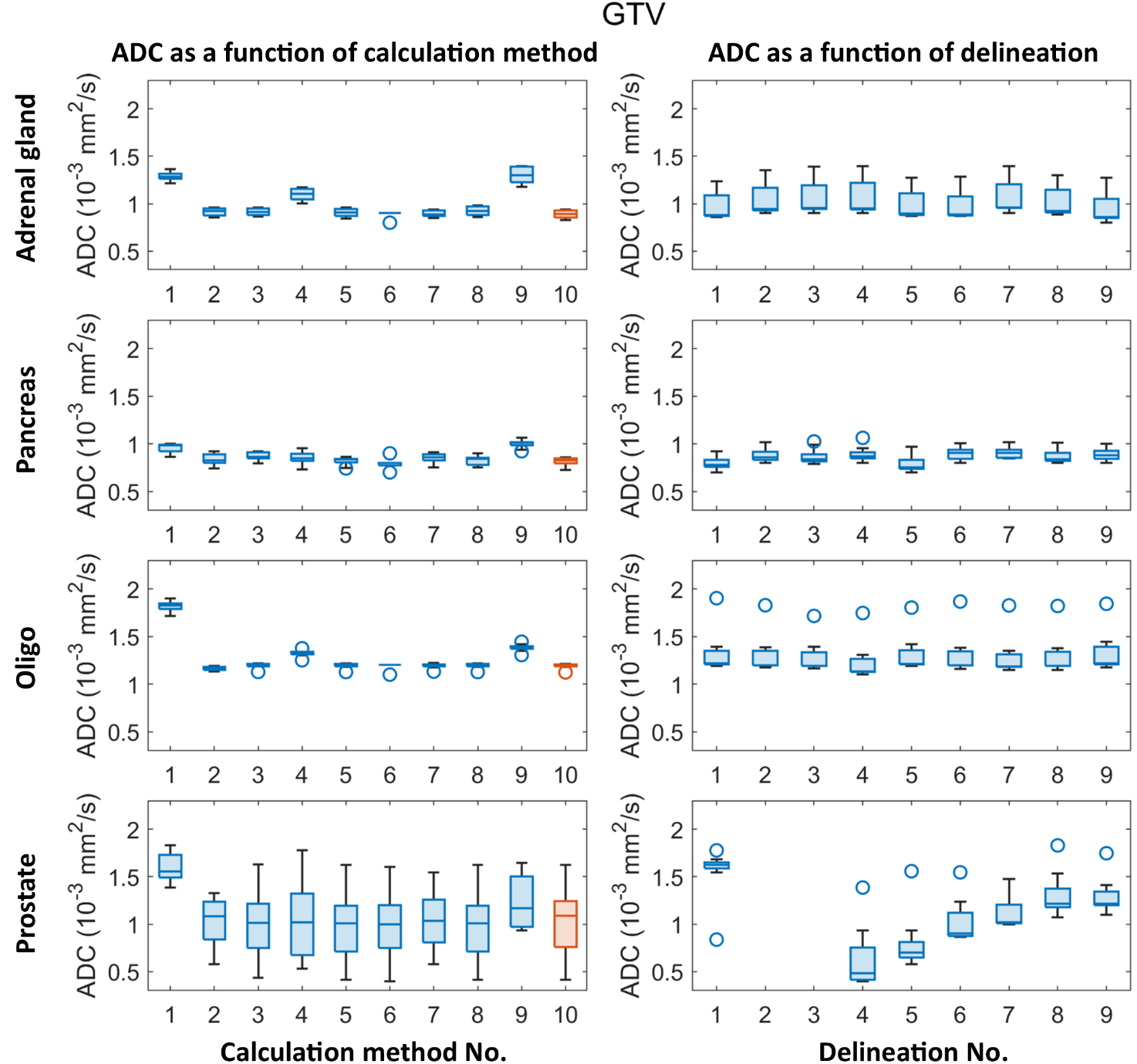


Figure S1: ADC values within GTVs as a function of calculation method (left) and delineation (right) for the four clinical cases. The boxes represent variation across delineations and calculation methods, respectively. For comparison, calculation method no. 10 (red) represent the scanner software using b-values$\geq$150mm/s^2^. The ‘o’ marker indicates outliers, defined as more than 1.5 times the interquartile range away from the bottom or top edges of the box.


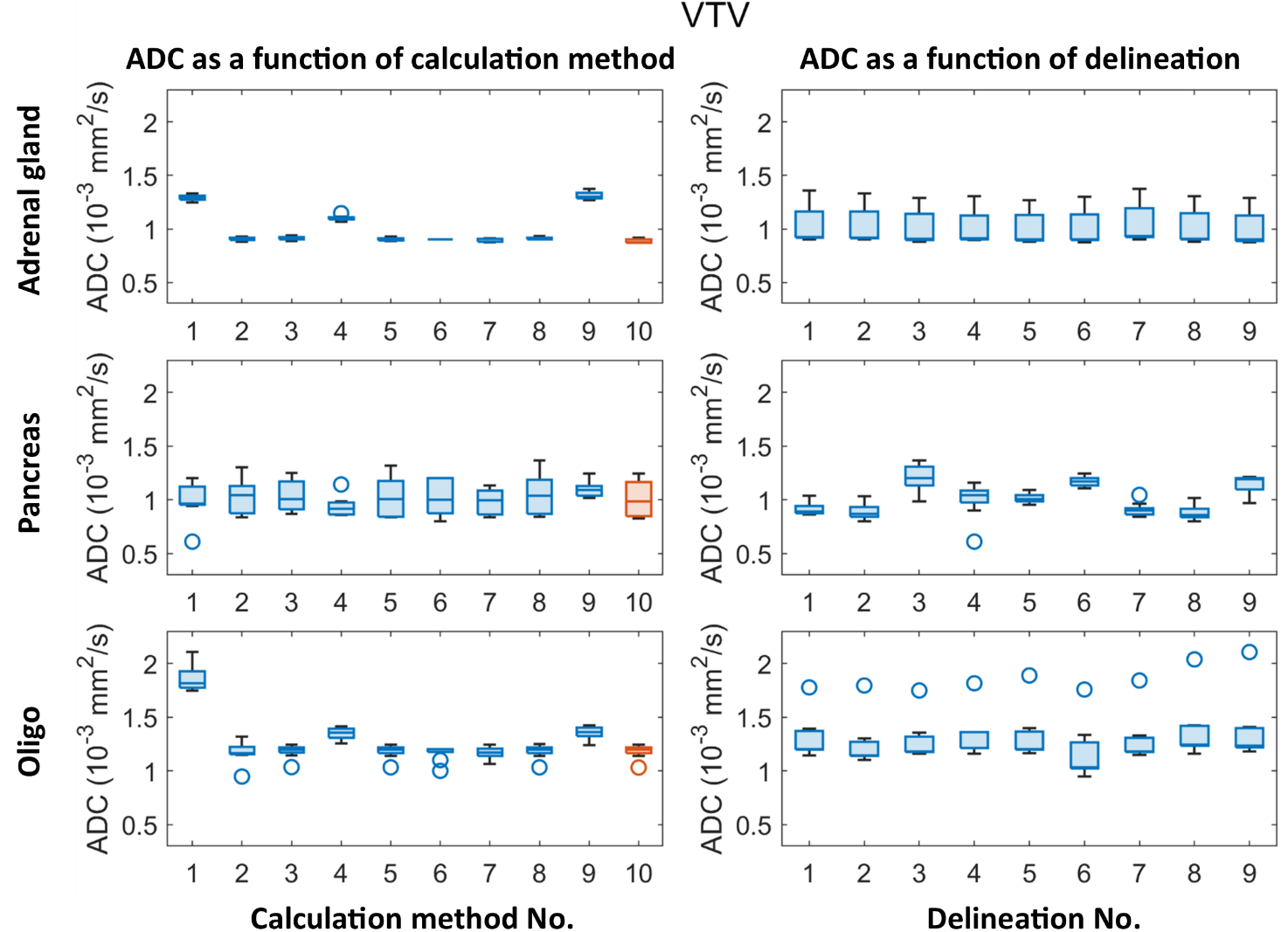


Figure S2: ADC values within VTVs as a function of calculation method (left) and delineation (right) for the four clinical cases. The boxes represent variation across delineations and calculation methods, respectively. For comparison, calculation method no. 10 (red) represent the scanner software using b-values$\geq$150mm/s^2^. The ‘o’ marker indicates outliers, defined as more than 1.5 times the interquartile range away from the bottom or top edges of the box.


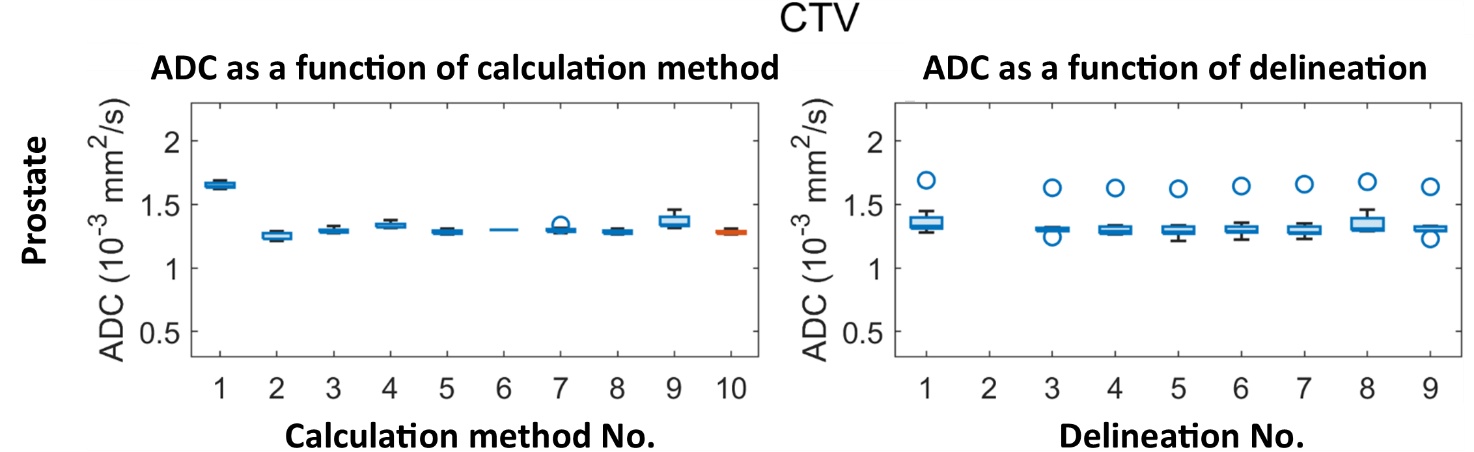


Figure C3: ADC values within CTVs as a function of calculation method (left) and delineation (right) for the prostate. The boxes represent variation across delineations and calculation methods, respectively. For comparison, calculation method no. 10 (red) represent the scanner software using b-values$\geq$150mm/s^2^. The ‘o’ marker indicates outliers, defined as more than 1.5 times the interquartile range away from the bottom or top edges of the box.

# Weighted least squares fitting

The Stejskal-Tanner model for the DWI signal, S, is given by [1]:

$$S=S_{0}\cdot e^{-b\cdot ADC}$$

By taking the logarithm on both sides, we get:

$$\ln\left( S \right)=\ln\left( S_{0} \right)-b\cdot ADC$$

For one voxel, measurements are performed for $N$ different b-values of the MRI signal magnitude. $S_{i}$ denotes the signal at the $i$-th b-value, measured with $n_{i}$ measurements (number of measurements = number of excitations (NEX)).

For each single measurement, we assume Gaussian noise, $\sigma$, that is independent on signal and b-value. Linear fitting is performed for the logarithm to the signal: $y_{i}=ln(S_{i})$. To determine the variation associated with $y_{i}$, we use uncertainty propagation. For a function of a single variable, f(x), the uncertainty propagation equation is given by:

$$\sigma_{f}^{2}=\left( \frac{df}{dx} \right)^{2}\sigma_{x}^{2}$$

Applying this to $y_{i}$, we get:

$$\sigma_{y_{i}}^{2}=\left( \frac{d_{y_{i}}}{d_{S_{i}}} \right)^{2}\sigma_{S_{i}}^{2}=\frac{\sigma_{S_{i}}^{2}}{S_{i}^{2}}$$

Assuming that the measurements are uncorrelated,

$$\sigma_{S_{i}}=\frac{\sigma}{\sqrt{n_{i}}}$$

Combining the above expressions,

$$\sigma_{y_{i}}^{2}=\frac{\sigma^{2}}{n_{i}S_{i}^{2}}$$

Thus, the weights used for the weighted least squares fitting are:

$$w_{y_{i}}=\frac{1}{\sigma_{y_{i}}^{2}}=\frac{n_{i}S_{i}^{2}}{\sigma^{2}}$$

Since we assume that $\sigma$ is constant over all measurements, it is left out:

$$w_{y_{i}}=n_{i}S_{i}^{2}$$

# References

[1] Stejskal EO, Tanner JE. Spin diffusion measurements: Spin echoes in the presence of a time-dependent field gradient. J Chem Phys. 1965;42:288–292.
